# Microwave tomography: review of the progress towards clinical applications

**DOI:** 10.1098/rsta.2009.0092

**Published:** 2009-08-13

**Authors:** Serguei Semenov

**Affiliations:** School of Medicine, ISTM, University of KeeleStoke-on-Trent ST4 7QB, UK

**Keywords:** microwave tomography, functional imaging, cardiac imaging, neuroimaging, malignancies

## Abstract

Microwave tomography (MWT) is an emerging biomedical imaging modality with great potential for non-invasive assessment of functional and pathological conditions of soft tissues. This paper presents a review of research results obtained by the author and his colleagues and focuses on various potential clinical applications of MWT. Most clinical applications of MWT imaging have complicated, nonlinear, high dielectric contrast inverse problems of three-dimensional diffraction tomography. There is a very high dielectric contrast between bones and fatty areas compared with soft tissues. In most cases, the contrast between soft-tissue abnormalities (the target imaging areas) is less pronounced than between bone (fat) and soft tissue. This additionally complicates the imaging problem. In spite of the difficulties mentioned, it has been demonstrated that MWT is applicable for extremities imaging, breast cancer detection, diagnostics of lung cancer, brain imaging and cardiac imaging.

## 1. Introduction

Current clinical tomographic imaging methods, including the three most widely used ones—computed tomography (CT), positron emission tomography (PET) and magnetic resonance imaging (MRI), each offer useful information on various tissue properties related, for example, to tissue blood perfusion, ischaemia and infarction, hypoxia, metabolism and malignancies. Each of these methods has its own advantages and disadvantages. For example, CT presents excellent spatial resolution, while is less informative in soft-tissue functional imaging than PET. On the other hand, PET cannot compete with CT in terms of spatial resolution. Recent trends in fused multimodality imaging have raised concerns about the costs of the systems and the dosage of radiation. There is a need for novel competitive imaging modalities. Microwave tomography (MWT) might present a safe, mobile and cost-effective supplement to current imaging modalities for non-invasive assessment of acute and chronic functional and pathological conditions of soft tissues. MWT was underdeveloped for a number of technical reasons, including the high costs of unique hardware components and an insufficient computing power. In recent years, tremendous progress in both telecommunication (mobile) technologies and computing has opened up unique opportunities for further research and development of MWT towards biomedical and clinical applications.

Within the microwave spectrum, biological tissues are differentiated and consequently can be imaged based on their dielectric properties. It is known that the dielectric properties of tissues with high (muscle) and low (fat and bone) water content are significantly different (Gabriel *et al*. 1996; Polk & Postow 1996). During recent decades, the changes in the dielectric properties of tissues caused by various physiological and pathological alterations have been intensively studied. These changes are summarized and discussed below.

Safety is an important feature of MWT imaging. Within this modality, a non-ionizing electromagnetic (EM) field is used in contrast to the ionizing radiation used in CT imaging and nuclear medicine. We estimate that the level of the microwave field used in imaging procedures will be comparable to the level of the microwave field used in cell phones at the same GHz portion of the EM spectrum.

This paper reviews our own results in areas of potential clinical applications of MWT imaging and presents our view on its state-of-the-art and potential clinical applications. Five initial potential areas of clinical applications of MWT were identified and reviewed here: extremities imaging, breast cancer detection, diagnostics of lung cancer, brain imaging and cardiac imaging. Of course, this is just the beginning of the list and is not conclusive in any sense. Both the technical aspects of MWT and imaging algorithms are outside the scope of this paper.

## 2. Extremities soft-tissue imaging

The successful management of a fractured bone involves an understanding of the two major components of any segments of injured extremity. These two components are the bony element and the soft-tissue elements (skin, muscle, nerve and vessels). The diagnosis and evaluation of the bony component are easily carried out by the treating physician using radiographic studies. The accurate assessment of the soft-tissue component of the injured segments remains a major deficiency in the management of fractures. MWT, being successful in assessment of soft-tissue conditions and combined with plain radiology in the acute emergency situation, would provide the treating surgeon with a complete assessment of both components of any given injury.

Microwave tomographic imaging of the extremities possesses a complicated, nonlinear, high dielectric contrast inverse problem of diffraction tomography. There is a very high dielectric contrast between bones and soft tissues in the extremities. This might help to image bones, but the goal is to image soft tissues. To additionally complicate the problem, the contrast between soft-tissue abnormalities is expected to be less pronounced (for obvious reasons) than between bone and soft tissue. However, there is an obvious simplification of the problem: some portions (segments) of the extremity can be two-dimensionally approximated. This allows for the use of a less complicated two-dimensional imaging approach when compared with three-dimensional imaging.

**Figure 1:**
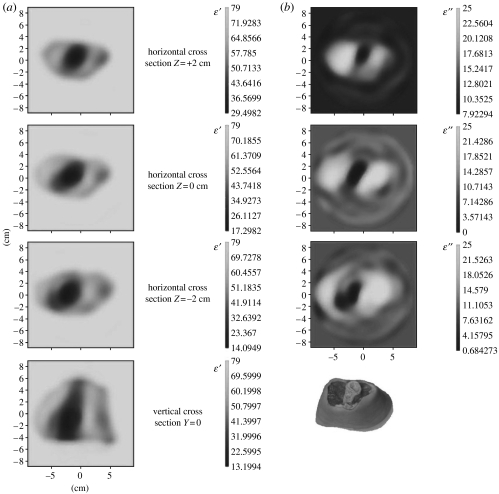
Reconstructed images of an excised segment of a pig hind leg for (*a*) *ϵ*′ and (*b*) *ϵ*″. Scales are in cm. Frequency 0.9 GHz. Reconstruction using a three-dimensional gradient approach. (Reproduced from the Biophysical Laboratory, Carolinas Medical Center, Charlotte, NC, USA.)

In order to access the applicability of MWT for extremities imaging, we have conducted experiments on an excised segment of a pig hind leg (Semenov *et al*. 2005). One of the successive three-dimensional images is presented in figure 1. Horizontal (+2 cm, 0 cm and −2 cm from the transmitter vertical position) and vertical (*Y*=0 cm) cross sections of the reconstructed images are presented for *ϵ*′ (figure 1*a*) and *ϵ*″ (figure 1*b*). Bone and soft tissue are clearly reconstructed. Note that the frequency used was 0.9 GHz, applicable for imaging larger objects, for example whole-body imaging. Higher frequencies (up to 3–4 GHz) are applicable (in terms of penetration) for extremity imaging, producing higher resolution images.

**Figure 2:**
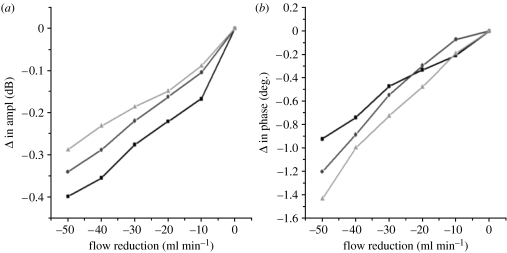
Changes in (*a*) amplitude and (*b*) phase of MW signal transmitted through pig thigh due to a reduction in femoral blood flow. Results of three series of flow reduction (squares, first flow reduction; circles, second flow reduction; triangles, third flow reduction).

In experiments conducted on pig skeletal muscle, we detected microwave signals passed through a pig thigh (Semenov *et al*. 2007). Changes in amplitude (figure 2*a*) and phase (figure 2*b*) of microwave signals transmitted through a pig thigh, due to reduction in femoral blood flow, are shown in figure 2. There were three series of short flow reductions (with duration approx. 2–3 min each) with 10 min ‘washout’ periods in between each series. An immediate change in microwave amplitude and phase was observed immediately after the flow reduction. A linear correlation of changes in blood flow and detected amplitude and phase of microwave signal was also observed. Some cumulative effect was observed—second- and third-flow reductions were usually less pronounced. The technology demonstrates very high sensitivity, being able to pick up flow reduction increments of 1–2 ml min^−1^.

**Figure 3:**
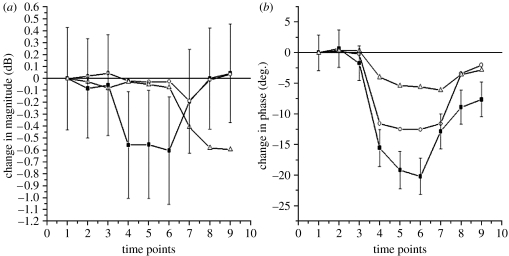
Changes in (*a*) amplitude and (*b*) phase of MW signal transmitted through a volunteer's forearm during a short occlusion of blood flow (time points 4, 5 and 6). Results of three series (squares, first set; circles, second set; triangles, third set) of intervention are shown with 10 min ‘resting’ periods in between each series.

The next series of blood flow reduction experiments were conducted on the forearms of human volunteers using an ethics committee-approved protocol. Forearm blood flow was compromised, briefly, by inflating a sphygmomanometer cuff to just above systolic blood pressure. Three series of short interventions were conducted with 10 min rest periods in between consecutive interventions. The results of the experiments are presented in figure 3 for one of volunteers. An immediate change in microwave signals (with more pronounced changes in its phase) was observed immediately after flow reduction. Again, some cumulative effect was observed—the second and third flow reductions were usually less pronounced.

**Figure 4:**
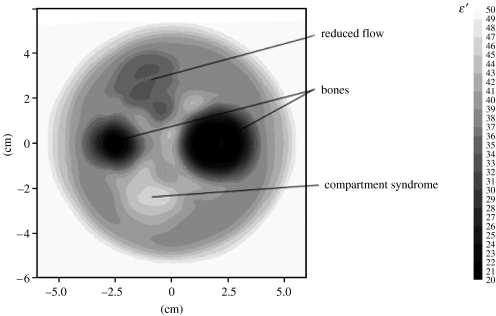
Simulated microwave tomographic image of pig thigh with areas of reduced blood flow and compartment syndrome. Frequency 2.0 GHz. Reconstruction using a two-dimensional Newton approach.

Further experiments were conducted on a pig thigh with acute compartment syndrome. The results demonstrated that the technology is sensitive for detection of compartment syndrome. Furthermore, computer simulations of a whole cycle of two-dimensional MWT images were conducted in order to assess the feasibility of the technology for imaging small (%) changes in soft tissues within a high-contrast dielectric object, observed in extremities with bones (Semenov *et al*. 2007). A reconstructed image of *ϵ*′ is presented in figure 4. The imaging results demonstrate areas of reduced blood flow and compartment syndrome.

## 3. Diagnostics of tissue malignancies

### (a) Malignancies and dielectric properties of tissue

The dielectric properties of normal and malignant tissues have been studied. Almost a century ago in 1926 Fricke and Morse reported that, at 20 kHz, the permittivity of malignant breast tumour is higher than that of normal breast tissue (Fricke & Morse 1926). In the 1980s and 1990s, it was demonstrated that the dielectric properties of malignant tumours and normal tissues are different in the breast (Joines *et al*. 1980, 1994; Chaudhary *et al*. 1984; Surowiec *et al*. 1988), liver (Smith *et al*. 1986; Joines *et al*. 1994) and lung (Marimoto *et al*. 1993; Joines *et al*. 1994).

#### (i) Breast tissues

The large contrast in dielectric properties (approx. 10 : 1) between normal and malignant breast tissues at microwave frequencies reported previously (Joines *et al*. 1980, 1994; Chaudhary *et al*. 1984; Surowiec *et al*. 1988) has generated an enthusiasm for researchers to focus on using microwaves for detection of breast tumours. To detect such a high-contrast tumour, various simplified, non-tomographic imaging approaches were suggested. However, the reported contrast in dielectric properties between normal and malignant breast tissues was studied in detail in the most recent large-scale study conducted by a collaborative research team from the University of Wisconsin and the University of Calgary (Lazebnik *et al*. 2007*a*,*b*). It has been pointed out that: (i) ‘there is a large variation in the dielectric properties of normal breast tissue due to substantial tissue heterogeneity’ (Lazebnik *et al*. 2007*a*) and (ii) the contrast in dielectric properties ‘between malignant and normal adipose-dominant tissues in the breast is considerable, as large as 10 : 1’, while the contrast ‘between malignant and normal glandular/fibroconnective tissues in the breast is no more than approximately 10 per cent’ (Lazebnik *et al*. 2007*b*). This highlights the need for development of sophisticated microwave imaging approaches that will be able to detect not only 10 : 1 contrast tumour, but also less pronounced 10 per cent differences in breast tissue dielectric properties.

#### (ii) Liver tissues

The most up-to-date study of *ex vivo* and *in vivo* normal and malignant liver tissues was reported in O'Rourke *et al*. (2007). It was concluded ‘that the dielectric properties of *ex vivo* malignant liver tissue are 19–30% higher than normal’, while the same differences in ‘*in vivo* tissues are not statistically significant’ with the exception of conductivity at 915 MHz.

#### (iii) Lung tissues

We have conducted a preliminary study of the dielectric properties of normal and malignant lung tissues using a rabbit model with VX-2 tumour (Semenov *et al*. 2003*a*). One example is shown in table 1. A suspension of homogenized VX-2 tumour was injected into the left lung lobe and the rabbit was restudied after two weeks of inoculation. Dielectric properties data for the left (injected) and right lobes of the lung are presented in table 1. Significant differences (contrast) in dielectric properties were observed. The results for *ϵ*′ are consistent with published data for lung tissues (Joines *et al*. 1994).

#### (iv) Blood perfusion, malignancies and dielectric properties

Tissue malignancies, having more intense metabolism than normal tissue, require an increase in blood supply obtained by additional vascularization. If an imaging modality can detect malignant tissue together with an assessment of local tissue blood supply, tissue hypoxia and infarction, then this gives an additional diagnostic power to the technology. MWT is such an imaging modality, which can indeed detect both malignancies and local blood perfusion. Experimental results summarizing the diagnostic possibilities of MWT for tissue blood perfusion are summarized in §5*a*.

### (b) Breast cancer

X-ray mammography has been and continues to be the most effective and widely used imaging technique for breast cancer detection. It has been shown in a number of screening programmes that X-ray mammography has helped to reduce breast cancer mortality (Zhou & Gordon 1989; Hurley & Kaldor 1992). Sabel & Aichinger (1996) pointed out that ‘X-ray mammography has some shortcomings in terms of sensitivity and specificity: on the one hand, 5–15% of breast cancers are not visualized mammographically’; and, on the other hand, ‘overall yield of breast cancers per number of breast biopsies recommended on the basis of screening mammograms ranges between 10 and 50 per cent’. These facts highlight a need for additional imaging modalities for breast cancer screening/detection.

A review of breast imaging techniques other than X-ray can be found in Kopans (1984), Grobhadern (1992) and Sabel & Aichinger (1996). These imaging modalities include: ultrasonography (both scanning modes and Doppler methods); thermography; electrical impedance; methods based on MRI; methods using X-ray radiation other than traditional mammography; imaging methods with radionuclides; PET; and other methods. Some novel, emerging methods have already been developed commercially, for example ultrasound and electrical impedance systems (Siemens, www.med.siemens.com/medroot/en/prod/diag/womens/prod/trans/prin/index.html).

There have been various proposed approaches to using microwaves for the detection of breast tumours, for example the confocal technique, UWB radar and two-dimensional and three-dimensional tomographic approaches (Hagness *et al*. 1999; Fear & Stuchly 2000; Meaney *et al*. 2000; Fear *et al*. 2002; Kosmas & Rappaport 2005).

**Figure 5:**
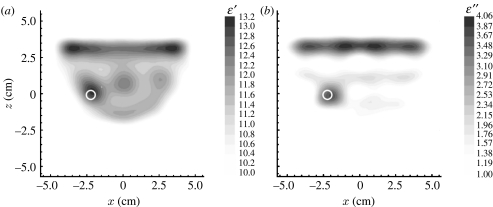
(*a*,*b*) Microwave tomographic mammography. Computer-simulated imaging of tumour with radius 3 mm. Frequency 3.5 GHz. A three-dimensional model consists of fatty breast with layers of muscle and bone on the top. The bright circle indicates the position of the tumour. Reconstruction using three-dimensional gradient method.

**Figure 6:**
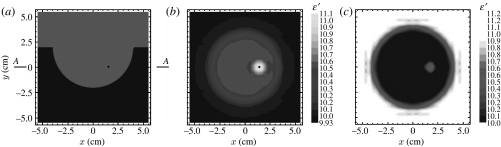
Microwave tomographic imaging of breast tumour. (*a*) Model, (*b*) results of reconstruction in cross section AA for a 2 mm tumour and (*c*) imaging results for a 1.5 mm tumour. Frequency 6 GHz. Reconstruction using a three-dimensional gradient method.

We studied the applicability of two approaches of using microwaves for breast cancer detection (Souvorov *et al*. 2000; Bulyshev *et al*. 2001). In Souvorov *et al*. (2000) we demonstrated the possibility of using a handheld two-dimensional microwave antenna array for the detection of superficial breast cancers. One of the results of the breast cancer imaging experiment using the three-dimensional MWT approach is shown in figure 5 (Bulyshev *et al*. 2001). Simulated three-dimensional MWT experiments were conducted at frequencies of 2.0, 3.5 and 6.0 GHz on a three-dimensional model of breast composed of a hemisphere of the breast itself (fatty tissue) and layers of muscle and bone on the top. The cancer was modelled as a small sphere with radius 3 mm immersed in the breast fatty tissue. Reconstructed images for both *ϵ*′ and *ϵ*″ clearly reveal a tumour area, as indicated with the bright circles into the images (figure 5). Further development of both three-dimensional scalar and three-dimensional vector image reconstruction algorithms would allow for significant improvement of tumour detectability with the help of MWT. The most recent images of 1.5 and 2 mm tumours are presented in figure 6. The reconstructed images (figure 6*b*,*c*) clearly reveal the small tumours.

### (c) Lung cancer

Lung cancer is the deadliest type of cancer in the USA and Europe. The American Lung Association estimated that in 2007 lung cancer was responsible for approximately 160 390 deaths, accounting for 28.7 per cent of all cancer deaths in the USA (American Cancer Society 2007), while simultaneously causing 213 380 new cases, accounting for almost 15 per cent of all cancer diagnoses (American Cancer Society 2007). These figures reflect that lung cancer continues to place a huge burden on the nation's health, more than cancers of the colon, breast and prostate combined. Survival rates for lung cancer tend to be much lower than those of most other cancers. The early diagnosis and precise staging are both desirable and crucial to lung cancer management. To achieve this, the optimal strategy should be accurate, non-invasive, safe and cost-effective imaging screening.

Each of the current imaging modalities used for lung screening and cancer detection (such as CT, MRI or PET) has its own advantages and weaknesses in the initial diagnosis and staging of lung cancer. MWT has the potential to provide an additional diagnostic power and present an effective supplement to current imaging modalities.

The challenging problem of lung imaging is in the detection of small malignant tumours within the highly dielectrically inhomogeneous and structurally complex human chest. In view of this, the imaging problem can be roughly divided into three subproblems: (i) *structural* imaging of a high dielectric contrast, large-scale biological object, (ii) *functional* imaging of small tissue inhomogeneities and (iii) detection of lung malignancies. Imaging results are devoted to address these problems.

**Figure 7:**
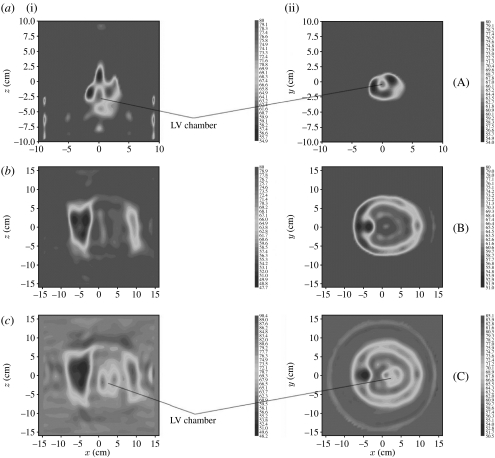
Reconstructed MWT images of (*a*) excised swine heart, (*b*) swine torso and (*c*) swine torso with heart for *ϵ*′. (i) Longitudinal view (*Y*=0) and (ii) transverse view (*Z*=0). Frequency 0.9 GHz. Scales are in cm. (Note the differences in scales between case (*a*) and cases (*b*) and (*c*).) LV, left ventricular chamber. Reconstruction using a three-dimensional gradient method.

Successive *structural imaging* of high dielectric contrast, large-scale biological objects is presented in figure 7 for: excised swine heart (figure 7*a*), swine torso (figure 7*b*) and swine torso with heart (figure 7*c*) for *ϵ*′. Three longitudinal views are presented in figure 7*a* and three transverse views are presented in figure 7*b*. Note the differences in scales between case (*a*) and cases (*b*) and (*c*) (Semenov *et al*. 2006). Overall, the images represent key anatomical features. In case (*a*) (the heart), the left ventricular (LV) chamber together with a shadow of the smaller right ventricular chamber is clearly seen. In case (*b*), the torso is successfully reconstructed, including the nicely reconstructed boundary of the chest cavity. Resuspension of the heart in the chest cavity reveals it on the images (case (*c*)—torso with heart). Importantly, the LV chamber and LV myocardial wall of the heart are clearly seen, in spite of shielding by the highly anatomically complicated and electromagnetically scattered thorax.

An example of the applicability of MWT for *functional imaging* is presented in §5, where the successive three-dimensional reconstruction of *ex vivo*, infarcted canine heart was achieved.

#### (i) MWT and lung filled with air

It has been suggested previously that since the lungs are filled with air, the potential for high reflections of EM radiation within the thorax could create a significant problem for microwave imaging. We measured dielectric properties of *in vivo* canine lung by the contact method with the help of a coaxial probe. The dielectric properties of intact lung largely differ from pure air and are *ϵ*=22 (±4)+*j*9 (±2) at a frequency of 0.9 GHz, which is consistent with the Federal Communication Commission tabulated values (FCC; Dielectric properties of body tissues at RF and microwave frequencies, http://www.fcc.gov/fcc-bin/dielec.sh). This can be understood since at this wavelength the average by volume dielectric properties are measured, including lung tissue, blood and air. This is also supported by the dependence of the dielectric properties of lung on the inhalation/exhalation phase of the respiratory cycle. For example, the dielectric properties of lung vary from 22+*j*8.5 (inflated) to 51+*j*16 (deflated) at 1 GHz (FCC).

**Figure 8:**
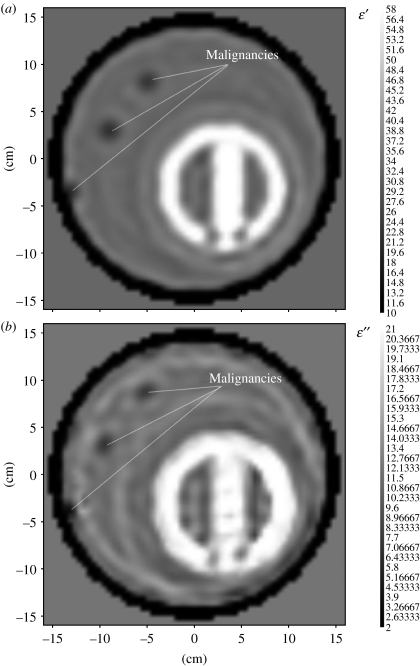
Reconstructed MWT images of a computer-simulated chest model with three lung ‘malignancies’ for (*a*) *ϵ*′ and for (*b*) *ϵ*″. Frequency 1 GHz. Scales are in cm. Reconstruction using a two-dimensional Newton method.

To assess the feasibility of the MWT imaging approach for detection of lung tumours, we further generalize our previously developed two-dimensional computer-simulated model of the chest (Souvorov *et al*. 1998). Three small, high-contrast inhomogeneities (*r*=7.5 mm) modelling lung malignancies were incorporated into different lung portions of the model (figure 8). Imaging results are presented in figure 8. In spite of a high dielectric contrast and spatial scale of the model, three ‘tumours’ were successfully detected. Of course, a simplified two-dimensional model does not cover all potential challenges of ‘real-life’ three-dimensional imaging cases. However, it does demonstrate the feasibility of the MWT imaging approach for detection of lung malignancies. Concluding this part, based on up-to-date knowledge, the differences in dielectric properties between normal and malignant lung tissues (contrast in dielectric properties) are approximately 10–15%. The detection of those relatively small differences within a high dielectric contrast and large-scale human torso is a very challenging problem. It has been demonstrated that the three-dimensional MWT approach is applicable for structural and functional imaging of large-scale, high-contrast biological objects, such as human thorax. An additional diagnostic power of microwave imaging technology might be based on its ability to assess blood supply, tissue hypoxia and infarction of metabolically active malignant tumours within the ms range of acquisition time, achievable by applying modern telecommunication technological advances to microwave tomographic imaging.

## 4. Brain imaging

According to the American Heart Association/Stroke Association, ‘every 45 s, someone in America has a stroke. Every 3 min, someone dies of one. Stroke killed an estimated 163 538 people in 2001 and is the nation's third leading cause of death, ranking behind diseases of the heart and all forms of cancer. Stroke is a leading cause of serious, long-term disability in the United States’, with significant economic impact: ‘in 2004, the estimated direct and indirect cost of stroke is $53.6 billion’ (American Heart Association/Stroke Association 2007).

Clinical management of stroke has been enhanced by the use of thrombolytics (clot busters) combined with the application of brain imaging techniques that reveal the pathophysiological changes in brain tissue that result from the stroke. In particular, the clinical decision to use a thrombolytic must be made within 3 hours of the onset of symptoms and requires a firm diagnosis of an ischaemic stroke (Adams *et al*. 2007). This clinical decision relies on imaging methods such as CT and MRI to reliably determine ischaemic perfusion changes. Subsequent management of the stroke is enhanced by imaging the extent of the area of brain tissue with compromised blood flow (Muir *et al*. 2006). Current clinical imaging methods, including CT, PET and MRI, each offer useful information on tissue properties related to perfusion, ischaemia and infarction (Muir *et al*. 2006). Each of these methods has its own advantages and disadvantages. While better understanding of stroke injury and efficiency of treatment obtained by repeated imaging will improve treatment of stroke patients, repeated CT or MRI head scans with perfusion and diffusion imaging would be necessary to inform clinical decisions. This would expose the patient repeatedly to radiation and contrast agents, and interfere with early rehabilitation. To date, there is no accurate and objective way to monitor recurrent ischaemia–reperfusion injury and the development of oedema repeatedly over periods of time without moving the patient.

A technique that is non-invasive, mobile, easily applied and could monitor the patient continuously in real time, either at the bedside or in the emergency department, would be a marked advance over existing imaging methods. MWT is a promising concept as it is a method that potentially offers non-invasive real-time, safe, mobile and cost-efficient tissue viability monitoring. MWT may also present an effective supplement to current imaging modalities for acute and chronic assessment of perfusion-related brain injuries.

**Figure 9:**
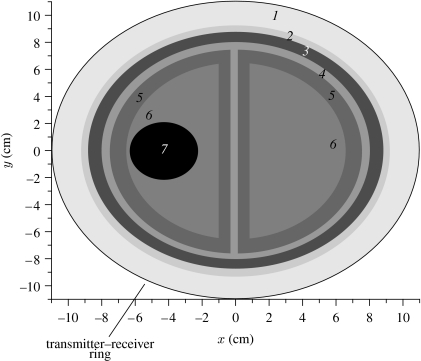
Simulated two-dimensional model of a head. Transmitters and receivers (equidistantly positioned) are located on the outer ring with *R*=11 cm. The working chamber is filled with matching solution (1) of eps=40+*j*13. The dielectric properties (at 1 GHz) of the regions of the model were taken from (Gabriel *et al*. 1996; Schmid *et al*. 2003; Peyman *et al*. 2007; FCC): (2) skin, 40+*j*11; (3) skull, 13+*j*2; (4) CSF, 57+*j*26; (5) grey matter, 50+*j*18; (6) white matter, 40+*j*15; and (7) stroke area, 36+*j*13 (simulated).

There was always a question of how much EM energy can penetrate through a human head. We conducted experiments to address this question using human cadavers. All activities were conducted under the terms of the Human Tissues Act and in line with Keele University's Research Governance policies. Experimentally measured and computer-simulated attenuations are summarized in table 2. The properties of the solution were chosen to have higher overall attenuation within an imaging setting to avoid an influence on the measured signal of (i) diffracted on head EM waves and (ii) reflected from chamber boundaries EM waves. Experimentally measured attenuation within an imaging setting with human head is approximately −82 dB for sample A and approximately −86.5 dB for sample B at a frequency of 0.81 GHz. Those attenuations can be confidently measured with modern MW measurement technologies. Of course, there is an issue of differences between dielectric properties of embalmed brain tissues and *in vivo* brain tissue. We conducted computer simulation using the head model (figure 9) with tabulated dielectric properties of tissues (Gabriel *et al*. 1996; Schmid *et al*. 2003; Peyman *et al*. 2007; FCC; model 1 in table 2) and those of embalmed brain tissues (model 2 in table 2) for the same experimental setting: antenna–antenna distance of 24 cm and frequency 0.81 GHz. The overall measured and simulated attenuations in solution alone are in good agreement: −97.8 and −99 dB correspondingly. The differences in attenuations between two models are approximately −28 dB. Given that the maximal expected attenuation within an imaging setting of 24 cm in diameter (including highly attenuated matching media) is approximately −114.5 dB (86.5+28) at 0.81 GHz, this is within the technical performance of modern MW-measurement devices. The IFBW might be increased allowing for a shorter data acquisition time when low-noise amplification is used.

**Figure 10:**
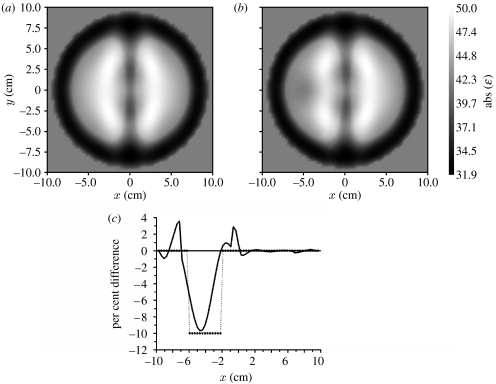
Reconstructed MWT images of a simulated brain model (figure 9): (*a*) normal and (*b*) with a stroke injury with radius 2 cm located at {−4, 0}; (*c*) the reconstructed differential profile (% difference) through the stroke area (squares, model image, solid curve, reconstructed image). Frequency 1 GHz. Reconstruction using a two-dimensional Newton method.

The brain imaging case presents a significant challenge, the brain being an object of interest located inside a high dielectric contrast shield, comprising the skull (13+*j*2) and cerebral spinal fluid (57+*j*26). However, high-performance, nonlinear inversion methods were able to produce biologically meaningful images, including images of stroke. One of the imaging results is presented in figure 10 for a frequency of 1 GHz with a 32×32 transmitters–receivers case. Normal brain image (figure 10*a*) is compared with an image with stroke (figure 10*b*) for the absolute values of the reconstructed dielectric properties. The reconstructed profile through the stroke area of radius 2 cm located at *X*=−4 cm and *Y*=0 cm is presented in figure 10*c* as the percentage difference between normal and stroke cases. These preliminary imaging results are not perfect. However, they indicate that MWT has the potential to determine perfusion-related changes in the human brain, and that MWT could be developed as a useful new imaging modality for stroke management.

**Figure 11:**
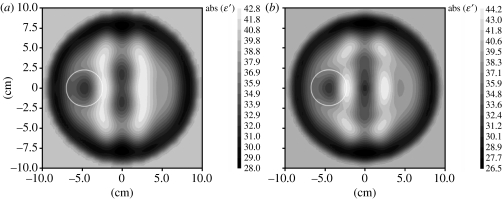
Reconstructed MWT images of a simulated brain model with a stroke injury with radius 2 cm located at {−4, 0} obtained using multi-frequency reconstruction: (*a*) 0.5 and 1.0 GHz; and (*b*) 2.0 and 1.0 GHz. 1% noise. Area with suspected stroke injury is circled in white. Reconstruction using a three-dimensional gradient method.

Further studies were conducted using multi-frequency imaging for practical noisy cases. Two successive imaging results are presented in figure 11. The first one (figure 11*a*) uses an initial imaging procedure at 0.5 GHz continuing at 1 GHz and the second one (figure 11*b*) uses initial inversion at 2.0 GHz continuing at 1 GHz. Both approaches demonstrate successful imaging results: the area of stroke injury (circled in white) has been reconstructed using both approaches.

Concluding this part, microwave tomographic imaging possesses great potential for functional neuroimaging and assessment of perfusion-related injuries within the brain. It has to be noted that MWT imaging might be applicable for fast (e.g. circulation-gated) imaging within the ms range of acquisition time.

## 5. Cardiac imaging

Coronary heart disease is the leading single cause of death in the industrial world (American Heart and British Heart Foundation Statistics, http://www.heartstats.org). Both MRI and CT have good spatial resolution but suffer from poor temporal resolution. Since MWT has the competitive advantage of very short frame rates/time resolution (within ms), it is expected that this technology might be used for more sophisticated analyses of cardiac function and viability of myocardial tissue.

In experimental studies, we have found that the dielectric properties of myocardial tissue have a strong dependence on coronary blood flow, myocardial hypoxia, acute ischaemia and chronic infarction (Semenov *et al*. 2000*a*, 2002). These findings are briefly reviewed below following MWT imaging results.

### (a) How myocardial conditions affect the dielectric properties

Experiments were conducted on dogs and pigs. All animal experiments were conducted using IACUC-approved protocols and NIH guidelines.

**Figure 12:**
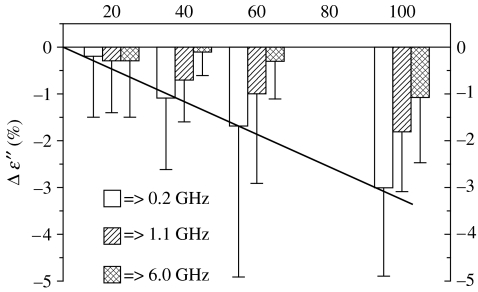
The changes in myocardial *ϵ*″ following 20, 40, 60 and 100% flow reduction. Mean data for a group of seven dogs in (%) from baseline values.

The changes in myocardial *ϵ*″ following 20 per cent, 40 per cent, 60 per cent and 100 per cent blood flow reduction are presented in figure 12 (Semenov *et al*. 2000*a*). This shows the mean relative change for a group of seven dogs, compared with mean baseline data, measured during a 15-min baseline period. As can be seen, a high degree of linear correlation was observed between changes in *ϵ*″ and the degree of occlusion: *R*=−0.997 at 0.2 GHz and *R*=−0.9987 at 1.1 GHz. A decrease in *ϵ*″ and, consequently, a decrease in myocardial conductivity most probably relates to a decrease in the integral value of blood, as a solution with a high degree of ion conductivity.

**Figure 13:**
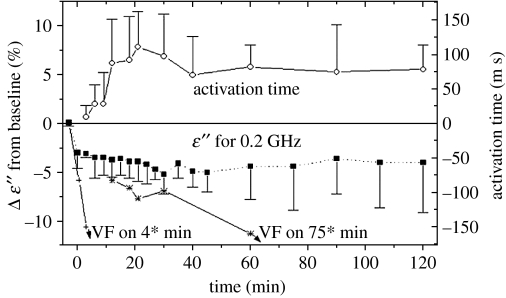
The changes in myocardial *ϵ*″ at 0.2 GHz following 2 hours of acute ischaemia. Mean data for a group of 10 dogs in (%) difference from baseline values.

The changes in the myocardial dielectric properties during 2 hours following total occlusion of the left anterior descending (LAD) artery are presented in figure 13; this shows the mean relative change in *ϵ*″ at 0.2 GHz for a group of 10 dogs, compared with mean baseline data measured during a 15 min control period (Semenov *et al*. 2000*a*). The change in electrical activation time (measured by local electrodes), as a marker of local ischaemia, is presented in the top portion of figure 13. The dielectric properties of myocardium changed during the first minute after total LAD occlusion. The changes stabilized at 15–60 min after occlusion, which is consistent with the development of irreversible myocardial injury. The large standard deviation from the mean seen in figure 12 may reflect intra-heart differences in the microwave probe position in relationship to the centre of the ischaemic zone, border zone and normal zone, and may also reflect differences in the epicardial collateral circulation between dogs.

**Figure 14:**
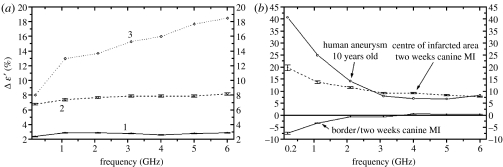
(*a*,*b*) The changes in myocardial dielectric properties in chronic two-week infarction. Mean data for a group of five canines in (%) difference from normal myocardium values. Values are compared with fresh tissue from a 10-year-old human post-infarction aneurysm (squares, border/two weeks canine MI; circles, centre of infarction/two weeks canine MI; diamonds, human aneurysm/10-years old).

The changes in the dielectric properties of chronic (two weeks) myocardial infarction are presented in figure 14 at a frequency range of 0.2–6.0 GHz (Semenov *et al*. 2000*a*). The changes are presented as mean data for a group of five dogs, relative to normal values of myocardial dielectric properties measured in the same dogs. Values are compared with fresh tissue from a 10-year-old human post-infarction aneurysm removed at surgery. Significant changes (up to 40%) were observed. It is significant that measurements of dielectric properties in these experiments can clearly delineate the infarcted zone from the border zone and normal myocardium. Note particularly *ϵ*″ near 1 GHz.

**Figure 15:**
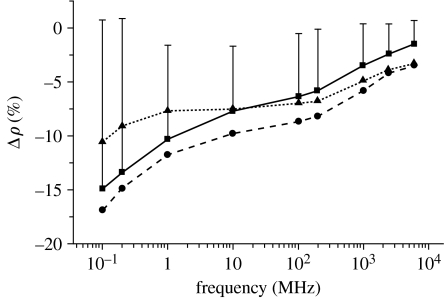
The spectral changes in myocardial resistance *ρ* during 10% hypoxia. The % difference from the mean baseline data (with s.d.), summarized for the group of seven dogs, is shown (squares, +9 min hypoxia; circles, +18 min hypoxia; triangles, +24 min hypoxia).

The spectral changes in myocardial resistance (*ρ*) during hypoxia are shown in figure 15 (Semenov *et al*. 2002). The percentage difference from the mean baseline data, summarized for a group of seven dogs, is shown. As can be seen from figure 15, hypoxia (10% for 30 min) decreases myocardial resistance (*ρ*). The decrease in bulk myocardial resistance is consistent with the results of experimental studies conducted using microelectrodes, which shows that hypoxia selectively decreases extracellular resistance.

### (b) MWT cardiac imaging results

**Figure 16:**
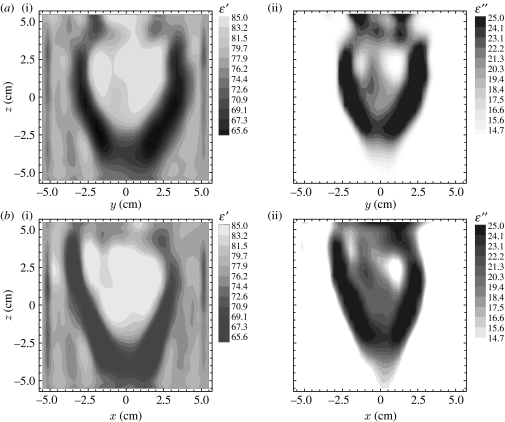
Reconstructed images (at 2.4 GHz) of explanted canine heart for (*a*(i),*b*(i)) *ϵ*′ and (*a*(ii),*b*(ii)) *ϵ*″. Two different vertical cross sections ((*a*) *X*=1.5 cm and (*b*) *Y*=1.5 cm) are shown. Reconstruction using a three-dimensional gradient method.

The reconstructed MWT image of an explanted canine heart is presented in figure 16. The experiments were conducted at 2.4 GHz using a full three-dimensional scheme of data acquisition and image reconstruction. Results are presented as two-dimensional vertical cross sections. The structure of the heart was successfully reconstructed, including both ventricular chambers (Semenov *et al*. 2000*b*).

**Figure 17:**
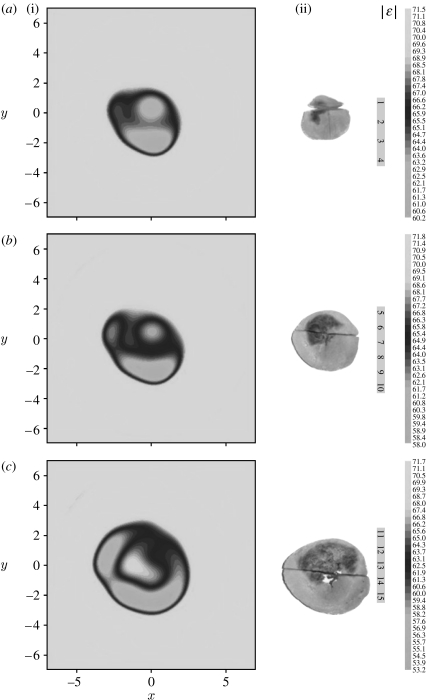
(*a–c*) Reconstructed electromagnetic tomography images of *ex vivo* infarcted canine heart: (i) transverse view for |*ϵ*| with an increment of image position in the longitudinal direction of 1 cm together with (ii) anatomical cross-sectional slices. Frequency 1 GHz. Scales are in cm. Reconstruction using a three-dimensional gradient method.

One example of the successive three-dimensional reconstruction of an infarcted canine heart is presented in figure 17 (Semenov *et al*. 2003*b*). Anatomical slices (figure 17*a*(ii)–*c*(ii)) demonstrate an area of left ventricle myocardium with significant infarction. The transverse images of |*ϵ*| together with corresponding anatomical slices are presented in figure 17*a*(i)–*c*(i) with an increment of image position in the longitudinal direction of 1 cm. The reconstructed images reveal shape and intra-heart structure. Furthermore, suspected areas of infarction (in terms of the dielectric properties) are consistent with anatomical slices. Therefore, in brief, it was demonstrated that MWT has the capability for both structural and functional cardiac imaging. The complicated and challenging problem of imaging in the thorax was addressed earlier in §3*c*.

## 6. Conclusions

MWT imaging is focused on non-invasive assessment of functional and pathological conditions of soft tissues. Most clinical applications of MWT imaging possesses a complicated, nonlinear, high dielectric contrast inverse problem of three-dimensional diffraction tomography. There is a very high dielectric contrast between bones and fatty areas compared with soft tissues. In most cases, the contrast between soft-tissue abnormalities (the target imaging areas) is less pronounced than between bone (fat) and soft tissue. This additionally complicates the imaging problem.

In spite of the difficulties mentioned, it has been demonstrated that MWT is applicable for extremities imaging, breast cancer detection, diagnostics of lung cancer, brain imaging and cardiac imaging.

## Figures and Tables

**Table 1:** Dielectric properties of *ex vivo* left (injected with VX-2) versus right lung lobes (mean±s.d.).

frequency (GHz)	1.0		2.45		3.0	
		
	*ϵ*′	*ϵ*′	*ϵ*′	*ϵ*′	*ϵ*′	*ϵ*′
left (injected)	51.4±2.7	20.2±1.1	49.8±2.5	15.0±0.9	49.2±2.5	15.6±0.8
right	59.5±2.8	22.2±1.1	58.0±2.9	16.6±0.7	57.3±2.9	17.1±0.5
*p*-value	0.004	0.03	0.004	0.02	0.003	0.02
contrast (%)	−13.6	−9.0	−14.1	−9.6	−14.1	−8.8

**Table 2:** Experimentally measured and computer-simulated attenuations (dB) through a head imaging setting (antenna distance=24 cm) filled with solution (80+*j*36). (Frequency 0.81 GHz, IFBW=10 Hz. Model 1, with tabulated dielectric properties of brain tissues (see the text) and geometry of figure 9. Model 2, with measured dielectric properties of brain tissues of sample (A): 45.4(±6.1)+*j*13.5(±2.7) at 0.81 GHz and geometry of figure 9.)

	experiment (dB)	model (dB)	
	
		1	2
solution (80+*j*36) only	−97.8	−99	−99
sample A in solution	−82.0	−98	−70
sample B in solution	−86.5		

## References

[bib1] Adams H.P. (2007). Guidelines for the early management of adults with ischemic stroke. Stroke.

[bib2] American Cancer Society 2007 Cancer facts and figures. See http://www.cancer.org/downloads/STT/CAFF2007PWSecured.pdf.

[bib3] American Heart Association/Stroke Association 2007 Heart disease and stroke statistics—2007 update. A report from the American Heart Association Statistics Committee and Stroke Statistics Subcommittee. See www.strokeassociation.org. Writing Group: Rosamond, W., Flegal, K. & G. Friday 2007 *Circulation* **115**, 69–171. 10.1161/CIRCULATIONAHA.106.179918 17194875

[bib4] Bulyshev A.E., Semenov S.Y., Souvorov S.Y., Svenson R.H., Nazarov A.G., Sizov Y.E., Tatsis G.P. (2001). Computational modeling of the three-dimensional microwave tomography of the breast cancer. IEEE Trans. BME.

[bib5] Chaudhary S.S., Mishra R.K., Swarup A., Thomas J.M. (1984). Dielectric properties of normal and malignant human breast tissues at radiowave and microwave frequencies. Indian J. Biochem. Biophys.

[bib6] Fear E.C., Stuchly M.A. (2000). Microwave detection of breast cancer. IEEE Trans. MTT.

[bib7] Fear E.C., Hagness S.C., Meaney P., Okoniewski M., Stuchly M. (2002). Enhancing breast tumor detection with near-field imaging. IEEE Microw. Mag.

[bib8] Fricke H., Morse S. (1926). The electrical capacity of tumors of the breast. J. Cancer Res.

[bib9] Gabriel S., Lau R.W., Gabriel G. (1996). The dielectric properties of biological tissues. II. Measurements in the frequency range 10 Hz to 20 GHz. Phys. Med. Biol.

[bib10] Grobhadern K. (1992). Nonmammographic breast imaging techniques. Curr. Opin. Radiol.

[bib11] Hagness S.C., Taflove A., Bridges J.E. (1999). Three-dimensional FDTD analysis of a pulsed microwave confocal system for breast cancer detection: design of an antenna-array element. IEEE Trans. AP.

[bib13] Hurley S.F., Kaldor J.M. (1992). The benefits and risks of mammographic screening for breast cancer. Epidemiol. Rev.

[bib14] Joines W.T., Jirtle R.J., Rafal M.D., Schaefar D.J. (1980). Microwave power absorption differences between normal and malignant tissue. J. Radiat. Oncol. Biol. Phys.

[bib15] Joines W.T., Zhang Y., Li C., Jirtle R.L. (1994). The measured electrical properties of normal and malignant human tissues from 50 to 900 MHz. Med. Phys.

[bib16] Kopans D.B. (1984). Early breast cancer detection using techniques other than mammography. Am. J. Roentgenol.

[bib17] Kosmas P., Rappaport C.M. (2005). Time reversal with the FDTD method for microwave breast cancer detection. IEEE Trans. MTT.

[bib18] Lazebnik M. (2007a). A large-scale study of the ultrawideband microwave dielectric properties of normal breast tissue obtained from reduction surgeries. Phys. Med. Biol.

[bib19] Lazebnik M. (2007b). A large-scale study of the ultrawideband microwave dielectric properties of normal, benigh and malignant breast tissues obtained from cancer surgeries. Phys. Med. Biol.

[bib20] Marimoto T., Kimura S., Konishi Y., Komaki K., Ugama T., Monden Y., Kinochi Y., Iritana T. (1993). A study of electrical bio-impedance of tumors. J. Invest. Surg.

[bib21] Meaney P.M., Fanning M.W., Li D., Poplack S.P., Paulsen K.D. (2000). A clinical prototype for active microwave imaging of the breast. IEEE Trans. MTT.

[bib22] Muir K.W., Buchan A., von Kummer R., Rother J., Baron J.-C. (2006). Imaging of acute stroke. Lancet Neurol.

[bib23] O'Rourke A.P., Lazebnik M., Bertram J.M., Converse M.C., Hagness S.C., Webster J.G., Mahvi D.M. (2007). Dielectric properties of human normal, malignant and cirrhotic liver tissue: *in vivo* and *ex vivo* measurements from 0.5 to 20 GHz using a precision open-ended coaxial probe. Phys. Med. Biol.

[bib24] Peyman A., Holden S.J., Watts S., Perrott R., Gabriel C. (2007). Dielectric properties of porcine cerebrospinal tissues at microwave frequencies: *in vivo*, *in vitro* and systematic variation with age. Phys. Med. Biol.

[bib12] Polk C., Postow E. (1996). Handbook of biological effects of electromagnetic fields.

[bib25] Sabel M., Aichinger H. (1996). Recent developments in breast imaging. Phys. Med. Biol.

[bib26] Schmid G., Neubauer G., Mazai P.R. (2003). Dielectric properties of human brain tissue measured less than 10h postmortem at frequencies from 800 to 2450 MHz. Bioelectromagnetics.

[bib27] Semenov S.Y., Svenson R.H., Tatsis G.P. (2000a). Microwave spectroscopy of myocardial ischemia and infarction. 1. Experimental study. Ann. Biomed. Eng.

[bib28] Semenov S.Y. (2000b). Three dimensional microwave tomography: experimental imaging of phantoms and biological objects. IEEE Trans. MTT.

[bib29] Semenov S.Y., Svenson R.H., Posukh V.G., Nazarov A.G., Sizov Y.E., Kassel J., Tatsis G.P. (2002). Dielectric spectroscopy of canine myocardium during ischemia and hypoxia at frequency spectrum from 100 kHz to 6 GHz. IEEE Trans. MI.

[bib30] Semenov, S. Y., Bulyshev, A. E., Posukh, V. G., Williams, T., Sizov, Y. E., Souvorov, A. E., Repin, P. N. & Voinov, B. A. 2003*a* Microwave tomography for detection of tissue malignancies: lung, liver and kidney sites. In *Progress in Electromagnetics Research Symposium, 2003, Honolulu, Hawaii, USA, 13–16 October*.

[bib31] Semenov S.Y., Bulyshev A.E., Posukh V.G., Sizov Y.E., Williams T.C., Souvorov A.E. (2003b). Microwave tomography for detection/imaging of myocardial infarction. 1. Excised canine hearts. Ann. Biomed. Eng.

[bib32] Semenov S.Y., Bulyshev A.E., Abubakar A., Posukh V.G., Sizov Y.E., Souvorov A.E., Van den Berg P., Williams T. (2005). Microwave tomographic imaging of the high dielectric contrast objects using different imaging approaches. IEEE Trans. MTT.

[bib33] Semenov S.Y., Posukh V.G., Sizov Y.E., Bulyshev A.E., Souvorov A.E., Nazarov A.G., Williams T.C., Repin P.N. (2006). Microwave tomographic imaging of the heart in intact swine. J. Electromagn. Waves Appl.

[bib34] Semenov S.Y., Kellam J.F., Althausen P., Williams T.C., Abubakar A., Bulyshev A., Sizov Y. (2007). Microwave tomography for functional imaging of extremity soft tissues: feasibility assessment. Phys. Med. Biol.

[bib35] Smith S.R., Foster K.R., Wolf G.L. (1986). Dielectric properties of VX-2 carcinoma versus normal liver tissue. IEEE Trans. BME.

[bib36] Souvorov A.E., Bulyshev A.E., Semenov S.Y., Svenson R.H., Nazarov A.G., Sizov Y.E., Tatsis G.P. (1998). Microwave tomography: a two-dimensional Newton iterative scheme. IEEE Trans. MTT.

[bib37] Souvorov A.E., Bulyshev A.E., Semenov S.Y., Svenson R.H., Tatsis G.P. (2000). Two-dimensional computer analysis of a microwave flat antenna array for breast cancer tomography. IEEE Trans. MTT.

[bib38] Surowiec A.J., Stuchly S.S., Barr J.R., Swarup A. (1988). Dielectrical properties of breast carcinoma and the surrounding tissues. IEEE Trans. BME.

[bib39] Zhou X., Gordon R. (1989). Detection of early breast cancer: an overview and future prospects. Crit. Rev. Biomed. Eng.

